# Local Cellular Immune Responses and Pathogenesis of Buruli Ulcer Lesions in the Experimental *Mycobacterium Ulcerans* Pig Infection Model

**DOI:** 10.1371/journal.pntd.0004678

**Published:** 2016-04-29

**Authors:** Miriam Bolz, Nicolas Ruggli, Nicole Borel, Gerd Pluschke, Marie-Thérèse Ruf

**Affiliations:** 1 Swiss Tropical and Public Health Institute, Basel, Switzerland; 2 University of Basel, Basel, Switzerland; 3 Institute of Virology and Immunology (IVI), Mittelhäusern, Switzerland; 4 Institute of Veterinary Pathology, University of Zurich, Vetsuisse Faculty, Zurich, Switzerland; Fondation Raoul Follereau, FRANCE

## Abstract

**Background:**

Buruli ulcer is a neglected tropical disease of the skin that is caused by infection with *Mycobacterium ulcerans*. We recently established an experimental pig (*Sus scrofa*) infection model for Buruli ulcer to investigate host-pathogen interactions, the efficacy of candidate vaccines and of new treatment options.

**Methodology/Principal Findings:**

Here we have used the model to study pathogenesis and early host-pathogen interactions in the affected porcine skin upon infection with mycolactone-producing and non-producing *M*. *ulcerans* strains. Histopathological analyses of nodular lesions in the porcine skin revealed that six weeks after infection with wild-type *M*. *ulcerans* bacteria extracellular acid fast bacilli were surrounded by distinct layers of neutrophils, macrophages and lymphocytes. Upon ulceration, the necrotic tissue containing the major bacterial burden was sloughing off, leading to the loss of most of the mycobacteria. Compared to wild-type *M*. *ulcerans* bacteria, toxin-deficient mutants caused an increased granulomatous cellular infiltration without massive tissue necrosis, and only smaller clusters of acid fast bacilli.

**Conclusions/Significance:**

In summary, the present study shows that the pathogenesis and early immune response to *M*. *ulcerans* infection in the pig is very well reflecting BU disease in humans, making the pig infection model an excellent tool for the profiling of new therapeutic and prophylactic interventions.

## Author Summary

Buruli ulcer is a necrotizing ulcerative disease of the skin and underlying tissue caused by infection with *Mycobacterium ulcerans*. Because patients often present late to health facilities, early stages of Buruli ulcer are only insufficiently described by histopathology. To study early host-pathogen interactions, we recently established an experimental pig infection model for Buruli ulcer. Here we used the model to study the pathogenesis and the local cellular immune responses upon infection with mycolactone-producing and non-producing *M*. *ulcerans* strains. Infection with toxin-producing bacteria led to the development of nodular lesions six weeks after infection, in which extracellular clumps of acid fast bacilli were surrounded by distinct layers of leukocytes. Ulceration of the nodular lesions subsequently led to the loss of most of the bacterial burden. In contrast, after infection with toxin-deficient *M*. *ulcerans* bacteria increased granulomatous cellular infiltration was observed, and massive tissue necrosis was absent. Pathogenesis as well as early immune responses to *M*. *ulcerans* infection in the pig is very well reflecting the human disease, making it a good model for the evaluation of the efficacy of new treatment options and candidate vaccines.

## Introduction

Buruli ulcer (BU) is a slow progressing, necrotising disease of the skin that mainly affects rural African communities [1,2] and is caused by *Mycobacterium ulcerans*. In contrast to other closely related mycobacterial pathogens such as *M*. *tuberculosis* and *M*. *leprae*, *M*. *ulcerans* produces a polyketide exotoxin named mycolactone, which is considered the main virulence factor of the bacteria and responsible for the extensive tissue damage seen in BU lesions [3,4]. Three distinct non-ulcerative forms of the disease are described (nodules/papules, plaques and oedema), which may all progress to ulceration as soon as the damage of the subcutaneous tissue leads to the collapse of the overlying epidermis and dermis [1].

Extensive histopathological analyses of advanced BU lesions were possible with excised tissue from surgically treated patients and diagnostic punch biopsies. The major hallmarks of *M*. *ulcerans* infection, which are also used for histopathological confirmation of clinical diagnosis, are the presence of coagulative necrosis, fat cell ghosts, epidermal hyperplasia and extracellular clusters of acid fast bacilli (AFB) in the absence of major inflammatory infiltrates in central parts of the lesions [5,6]. Although it was long thought that inflammatory infiltrates were completely absent in BU lesions [7], more recent studies demonstrated that cellular infiltration occurs at the periphery of lesions, where mycolactone levels are believed to be low [8,9]. The distribution of AFB and of cellular infiltrates is very heterogeneous in advanced BU lesions [7,10–12]. In the course of antibiotic treatment, massive leukocyte infiltration is observed, which culminates in the development of ectopic lymphoid structures in the lesions [13].

Since 2004, with the replacement of surgical treatment by the antibiotic combination therapy of rifampicin and streptomycin for eight weeks [14,15], tissue samples are no longer available for histopathological investigation. Additionally, the unknown mode of transmission, the very slow growth rate of *M*. *ulcerans*, and late care seeking behaviour of the affected populations are all factors that have made histopathological description of early BU stages difficult. This gap can be filled with the pig (*Sus scrofa*) model for experimental *M*. *ulcerans* infection we established recently [16], enabling the study of early host-pathogen interactions and pathogenesis in BU. Productive *M*. *ulcerans* infection in the pig skin leads to the development of lesions that closely resembled human BU lesions in their macroscopic as well as microscopic appearance [16]. All key features of BU pathology in humans were also found in the infected pig skin, which led us to conclude that the pig model is suitable for studying the early pathogenesis of BU and for the evaluation of new treatment and vaccination approaches [16].

In order to further characterize the developing lesions in the pig skin and in particular the role of mycolactone in the pathogenesis of BU, we aimed at determining the different infiltrating cell types by immunohistochemistry (IHC). To this end, protocols were established for different cell markers by IHC on formalin fixed, paraffin-embedded pig skin tissue. These markers were then used to characterize the cellular infiltrates in nodular and ulcerative lesions of the pig skin six weeks after infection. Finally, the lesions caused by wild-type *M*. *ulcerans* were compared immunohistochemically with lesions caused by mycolactone non-producing *M*. *ulcerans* strains.

## Material and Methods

### Ethical statement

All animal experiments were approved by the Animal Welfare Committee of the Canton of Berne under licence number BE92/14, and conducted in compliance with the Swiss animal protection law (SR 455).

### Bacteria

The *M*. *ulcerans* strain S1013 was isolated in 2010 from a swab taken from the undermined edges of the ulcerative lesion of a Cameroonian BU patient [17]. Two passages of the strain after isolation were done in BacT/ALERT medium (MB-251011, Biomerieux, USA) at 30°C. The mycolactone-deficient *M*. *ulcerans* strain S1228 was kindly provided for this study by Kris Huygen [18,19]. For preparation of the infection inocula, bacteria were cultivated in BacT/ALERT medium for nine weeks, recovered by centrifugation and diluted in sterile phosphate-buffered saline (PBS) to 375 mg/ml wet weight corresponding to 1.3 x 10^7^ colony forming unit (CFU)/ml (S1013) and 3.1 x 10^7^ CFU/ml (S1228) respectively, as determined by plating serial dilutions on 7H9 agar plates.

### Infection

Three specific pathogen-free 2-month-old pigs (Large White) from the in-house breeding unit of the Institute of Virology and Immunology (IVI) were kept under Biosafety-level-3 (BSL3) conditions one week prior and during the time of experimental infection. Animals were checked once daily for macroscopic signs of infection, had *ad libitum* access to water, straw and hay, and were fed daily with complete pelleted food.

Pigs were infected subcutaneously on both flanks at four to five infection sites with 1.3 x 10^6^ (six replicates on one pig) and 1.3 x 10^5^ (14 replicates on two pigs) CFU S1013 and 3.1 x 10^6^ (two replicates on one pig) and 3.1 x 10^5^ (four replicates on two pigs) CFU S1228 in 100μl PBS. Injection areas were wiped with 70% ethanol and bacterial suspensions injected subcutaneously with a 23G needle. Individual infection sites were encircled with a marker and the labelling renewed at least once a week. The pigs were euthanized at six weeks post-infection and tissue samples taken as described below.

### Euthanasia and necropsy

Pigs were euthanized by intravenous injection of pentobarbital (150 mg/kg bodyweight) and subsequent exsanguination. Skin tissue at infection sites were extensively excised with a scalpel and scissors, including all layers of the skin, the fascia and the first layer of muscle. The samples were transferred immediately into 10% neutral-buffered formalin solution (approx. 4% formaldehyde; HT501128-4L, Sigma-Aldrich, USA). Additionally to the infected skin tissue a total of 16 lymph nodes were excised. The three main draining lymph nodes (*Nll*. *cervicales superficiales ventrales and dorsales*) and the *Nll*. *Subiliaci* [20] were excised bilaterally from each pig, except for pig number two, for which only the *Nll*. *cervicales superficiales dorsales* and *Nll*. *subiliaci* were collected.

### Histopathological analysis

After fixation in formalin for 60 hours, samples were transferred to 70% ethanol for storage and transport, dehydrated and embedded into paraffin. 5 μm thin sections were cut, deparaffinised, rehydrated and directly stained with Haematoxylin/Eosin (HE; 51275-500ML, Sigma-Aldrich, Switzerland; 3446, J.T. Baker, Netherlands) or Ziehl-Neelsen/Methylene blue (ZN; 21820-1L, Sigma-Aldrich, Switzerland; 03978-250ML, Sigma-Aldrich, Germany) according to WHO standard protocols [1].

In order to characterize cellular infiltrates occurring in nodular and ulcerative lesions in the pig skin after infection with *M*. *ulcerans*, we conducted a search for antibodies to stain neutrophils, macrophages/monocytes, B-cells and T-cells. We preferentially tested monoclonal antibodies (mAb) against porcine antigens that were evaluated for IHC on paraffin-embedded tissue. Because of the high similarity of epitopes of human and pig cell surface molecules we also tested mAb against human antigens if no suitable reagents against the porcine antigens were found. Standard IHC staining protocols could be established for macrophages/monocytes, T-cells and neutrophils (Table 1).

**Table 1:** Antibodies for immunohistochemistry on pig skin.

Name	Specific for	Clone	Company	Catalogue number	Host species	Antigen retrieval method	Dilution
IBA-1	Macrophages, Microglia	polyclonal	Wako chemicals	019–19741	Rabbit	Citrate	1:2000
CD3	T-cells	CD3-12	Biorad	MCA1477	Rat	Citrate	1:100
CD107a	Macrophages, monocytes, granulocytes	4E9/11	Biorad	MCA2315GA	Mouse	Citrate	1:1000
Porcine neutrophils	Neutrophils	PM1	BMA Biomedicals	T-3503	Mouse	Citrate	1:200

**Fig 1:**
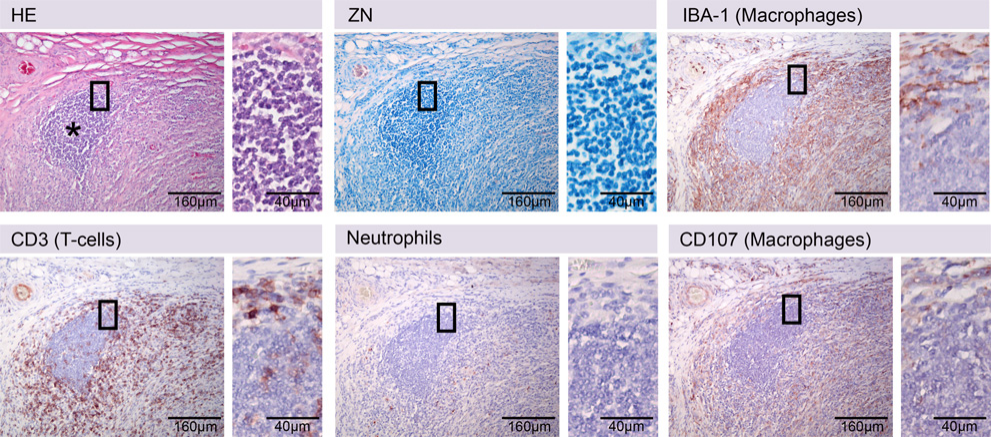
Established immunohistochemical stainings for leukocytes in the pig skin. Serial histologic sections of pig skin stained with Haematoxylin/Eosin (HE), Ziehl-Neelsen/Methyleneblue (ZN) and by immunohistochemistry protocols established for antibodies specific for T-cells (CD3), macrophages (IBA-1, CD107a) or neutrophils (21 kDa neutrophil protein). A B-cell cluster is shown that can only be identified by exclusion criteria (asterisk). The cluster of cells (asterisk) was not stained positive with antibodies against T-cells (CD3), macrophages (IBA-1, CD107a) nor neutrophils (Neutrophils), while the cells have lymphocyte appearance (ZN, insert).

Despite major efforts, labelling of B-cells was unsuccessful. Nevertheless, based on labelling of all other markers and exclusion of the non-labelled cells with lymphocyte appearance in HE and Methyleneblue staining, clusters of B-cells could be identified (Fig 1). For monocytes/macrophages, two markers were detected by IHC (IBA-1, CD107a), but due to stronger labelling (Fig 1), IBA-1 staining was preferred.

For IHC labelling, endogenous peroxidase was blocked in 0.3% H_2_O_2_ (31642-500ML, Sigma-Aldrich, Germany) for 20 min and unspecific binding was prevented by incubation with blocking serum matching the secondary antibody host. Subsequently, slides were pre-treated by the Citrate method [21] and incubated at room temperature for 1 hour with monoclonal antibodies in appropriate dilutions (Table 1). Sections were washed three times in PBS and incubated for 30 min with a biotinylated secondary antibody corresponding the primary antibody host (Goat-anti-rabbit, BA-1000; rabbit-anti-rat, BA-4001, both Vector Laboratories; goat-anti-mouse 1038–08, Southern Biotech). Sections were washed again three times in PBS and incubated for additional 30 min with streptavidin-horseradish peroxidase conjugate (VectastainR Elite ABC KIT; PK-6100, Vector Laboratories, USA). Staining was performed using Vector R NovaRed TM Substrate KIT (SK-4800, Vector Laboratories, USA) as a substrate and Meyer’s Haematoxylin as counterstain (Sigma). Stained sections were mounted with Eukitt mounting medium (03989-100ML, Sigma-Aldrich, Germany). Pictures were taken with a Leica DM2500B microscope or with an Aperio scanner at 20x magnification.

## Results

### AFB in the core of nodular lesions are surrounded by layers of infiltrating leukocytes

**Fig 2:**
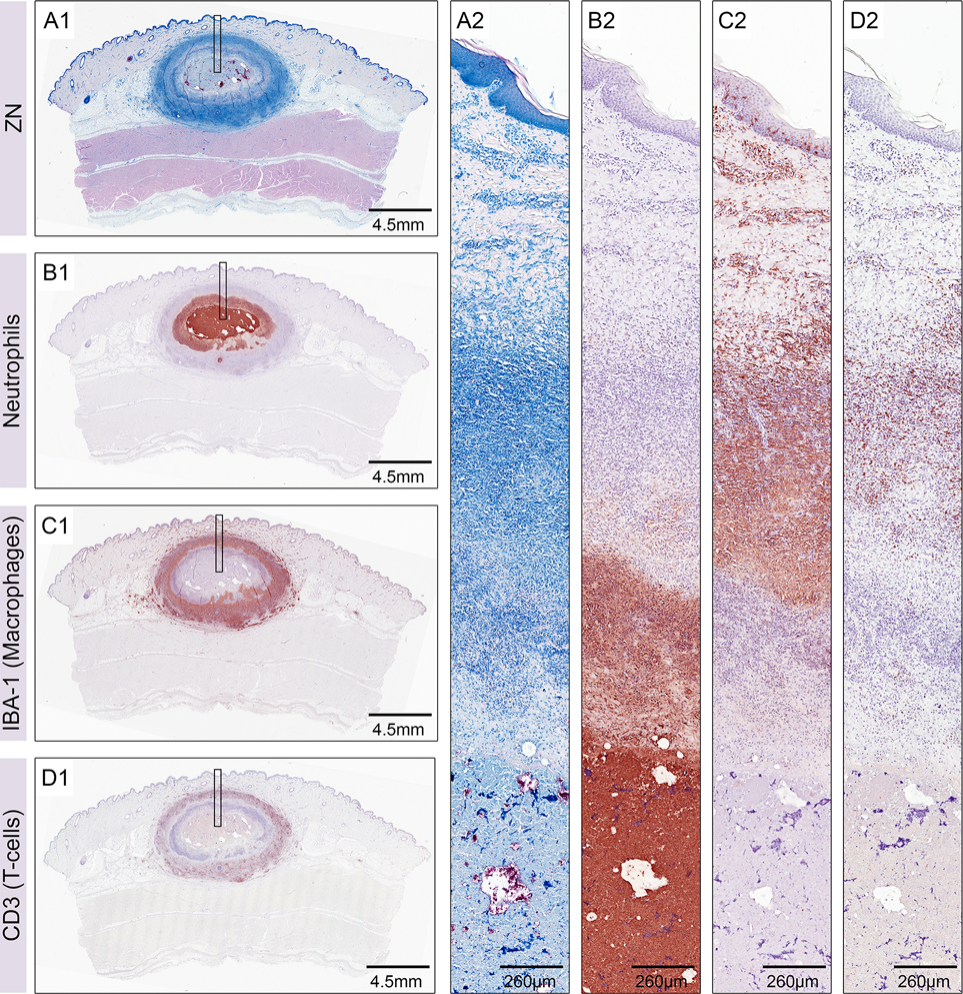
Layers of leukocyte infiltration in nodular lesions six weeks after infection. Histological sections of a nodular lesion six weeks after infection with 1.3 x 10^6^ CFU of *M*. *ulcerans*. Ziehl-Neelsen/Methyleneblue (ZN) staining revealed a strong cellular infiltration around a central necrotic core containing AFB stained in pink (A1, A2). The necrotic core and the surrounding ring of infiltration consisted of neutrophils (B1, B2). Neutrophils were surrounded by a belt of macrophages (C1, C2) that were heavily interspersed with T-cells (D1, D2).

Pigs infected with a dose of 1.3 x 10^6^ CFU *M*. *ulcerans* S1013 developed large nodular lesions of which 3/6 had ulcerated after six weeks. The central necrotic core of non-ulcerated lesions contained large clumps of AFB and was surrounded by layers of cellular infiltrates (Fig 2A1 and 2A2). What we previously defined as rings one, two and three [16] based on the degree of necrosis and cell types visible in Methyleneblue staining (S1 Fig), was now confirmed by IHC to be neutrophilic infiltration (Fig 2B1 and 2B2). The antibody used for staining of neutrophils does not only stain intact cells but also the remaining target antigen, a 21 kDa protein expressed by porcine neutrophils, in otherwise necrotic tissue. Strong staining of the completely necrotic centre of the lesion containing clumps of AFB (Fig 2B1 and 2B2) was therefore reflecting the presence of large amounts of neutrophilic debris. The next ring (previously described as ring number two [16]) consisted of apoptotic vesicles and neutrophils that were mostly necrotic, but retained some cellular appearance. Ring number three consisted mainly of intact neutrophils. These three rings were surrounded by a macrophage belt (Fig 2C1 and 2C2) that was interspersed with T-cells (Fig 2D1 and 2D2). T-cells appeared regularly distributed around the lesion and were more frequent towards the outside of the macrophage belt. Some of the lesions showed lymphocyte clusters at the border of the outermost infiltration ring that were CD3 negative and shared features with B-cell clusters found in human lesions [8,13,22].

**Fig 3:**
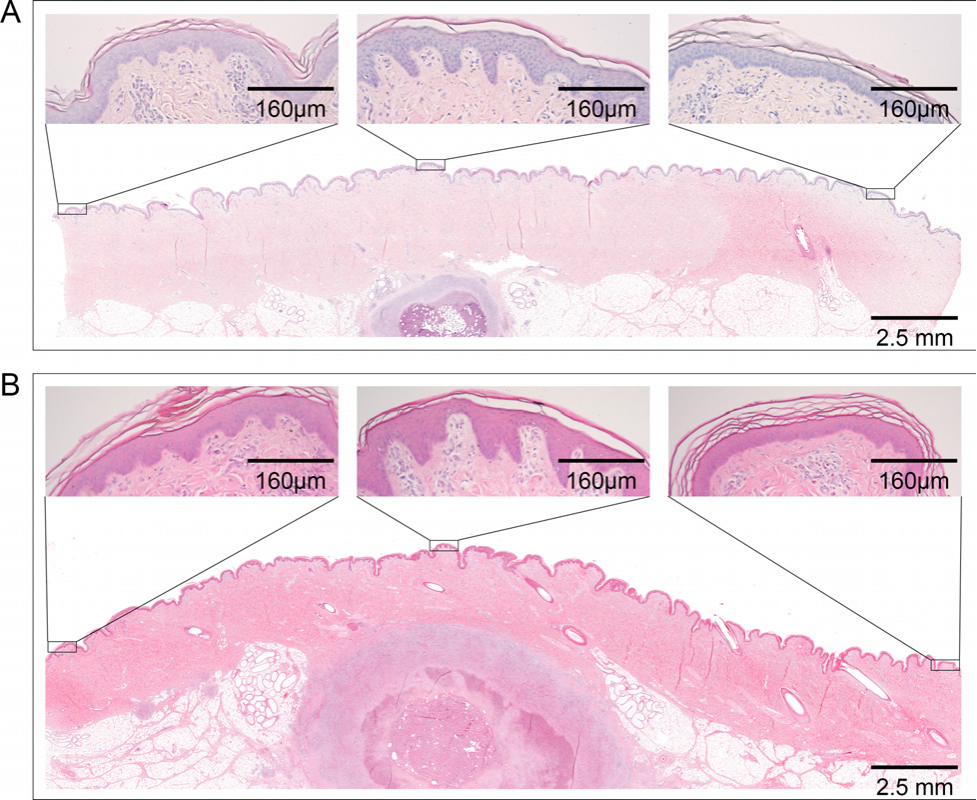
Epidermal hyperplasia above nodular lesions. Histological sections of nodular lesions stained with Haematoxylin/Eosin. (A) Small nodule caused by infection with 1.3 x 10^5^ CFU *M*. *ulcerans*. (B) Large nodule caused by infection with 1.3 x 10^6^ CFU *M*. *ulcerans*. Epidermal hyperplasia was strongest directly above the lesion (central box) and weaker if further away from the lesion.

Overall smaller nodules had developed six weeks after infection with a ten times smaller inoculum (i.e. 1.3 x 10^5^ CFU *M*. *ulcerans* S1013, Fig 3A). However, the general organization of the cellular infiltration was comparable to that of the lesions that had developed after inoculation with the higher dose (Fig 3B). Both small and large nodules displayed typical histopathological signs of BU found in human lesions, such as coagulative necrosis, extracellular clumps of AFB, fat cell ghosts and epidermal hyperplasia. While laterally to the main necrotic core and surrounding infiltration the epidermis appeared largely unchanged and showed no hyperplasia, the epidermis located above the lesion displayed clear thickening and elongation of the rete ridges (Fig 3).

### AFB containing necrotic tissue is sloughing off during ulceration

**Fig 4:**
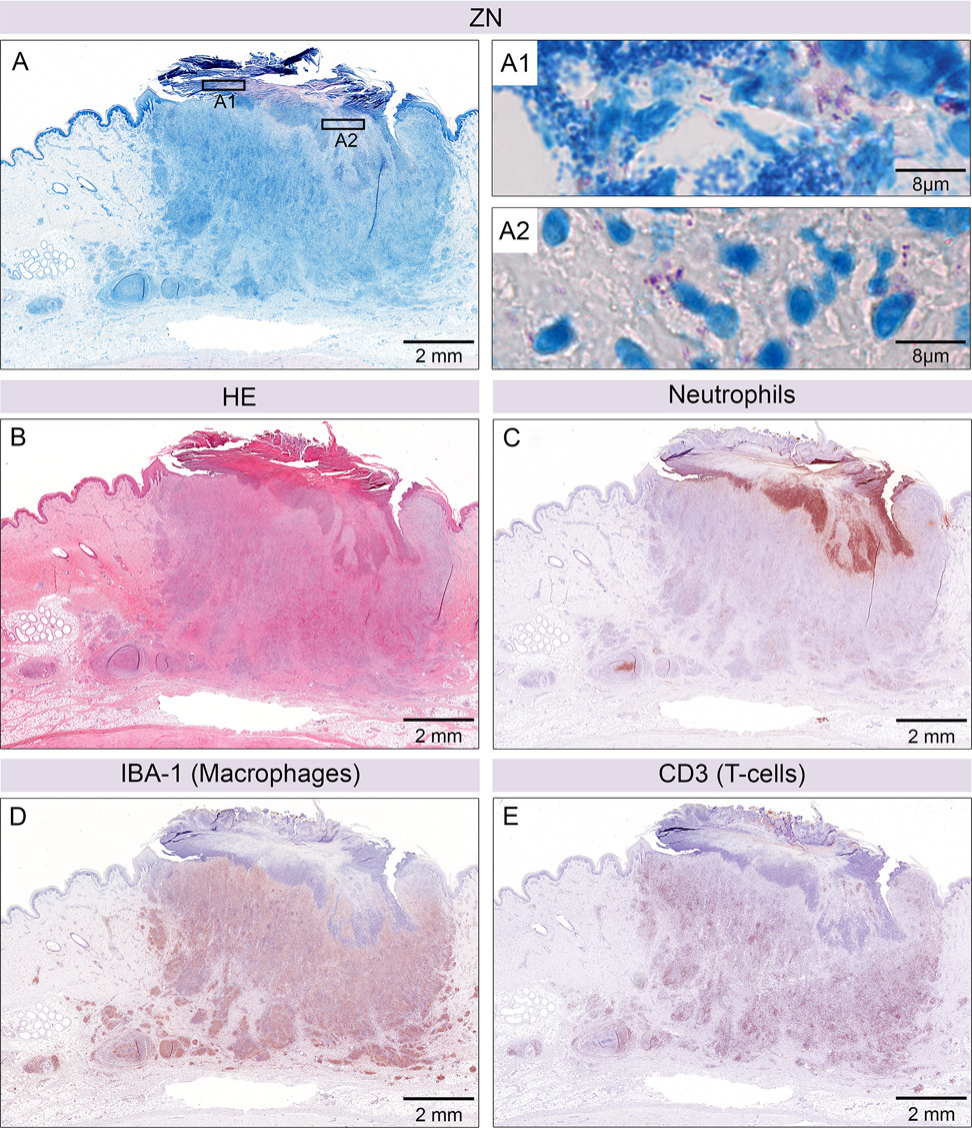
Ulcerative lesion after six weeks of infection. Histological sections of an ulcerative lesion six weeks after infection with 1.3 x 10^6^ CFU *M*. *ulcerans*. Ziehl-Neelsen/Methyleneblue (ZN) staining revealed AFB and bacterial debris in the crust (pink, A1) together with secondary infection (cocci, small blue dots, A1). More AFB were found just below the opening of the ulcer (A2). Haematoxylin-Eosin staining of the whole lesion (B). Neutrophils were predominantly located just below the opening of the ulceration (C) and in an additional focus in the subcutaneous fat layer. The majority of cellular infiltration consisted of macrophages (D) interspersed with a high number of T-cells (E).

Six weeks after infection with 1.3 x 10^6^ CFU *M*. *ulcerans* S1013, 3/6 lesions had ulcerated. IHC staining demonstrated that the necrotic core containing neutrophilic debris and the majority of AFB present in nodular lesions had been ejected through the opening in the epidermis (Fig 4A and 4C). Leftovers of the initially high bacterial burden were found in the crust that had remained on the surface of the ulcerative lesion (Fig 4A1) and in the neutrophilic infiltrate directly below the crust (Fig 4A2), representing also leftovers of the former necrotic core. Besides these residual AFB, colonization of the wound with cocci was found (Fig 4A1). The cellular infiltration that remained in the dermis between the fibroblasts consisted primarily of macrophages that were heavily interspersed with T-cells (Fig 4D and 4E).

### Infection with mycolactone-negative *M*. *ulcerans* leads to a granulomatous response

**Fig 5:**
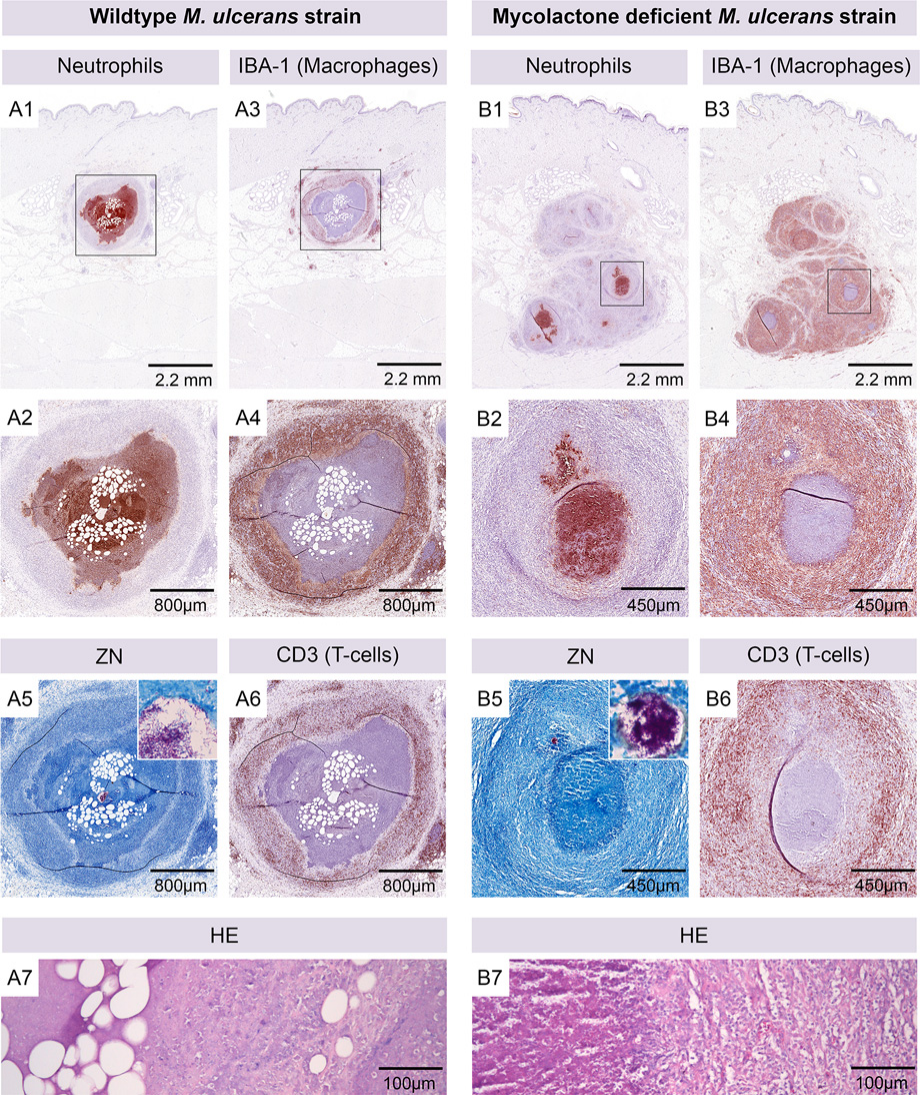
Different histopathological appearance of lesions caused by mycolactone producing and non-producing *M*. *ulcerans* strains. Histological sections of a nodular lesion six weeks after infection with 1.3 x 10^5^ CFU wild-type *M*. *ulcerans* (A). The organisation of cellular infiltration around a necrotic core containing AFB (A5) and fat cell ghosts (A7) was comparable to lesions caused by higher doses of *M*. *ulcerans*. The central part of the nodule contained neutrophils (A1, A2), surrounded by a ring of macrophages (A3, A4) that was interspersed with T-cells (A6). Infection with 3.1 x 10^5^ CFU of mycolactone-deficient *M*. *ulcerans* led to the development of a lesion with more inhomogeneously organized infiltration (B). Several granulomatous structures contained small cores consisting of neutrophils (B1, B2) and AFB (B5). Macrophage infiltration was massive (B4) and interspersed with numerous T-cells (B6). Fat cell ghosts were absent and necrosis was less extensive (B7).

In order to assess how the production of the cytotoxic macrolide exotoxin mycolactone affects the local cellular infiltration we infected pigs with a mycolactone-deficient *M*. *ulcerans* mutant and compared the resulting lesions to those caused by wild-type *M*. *ulcerans* bacteria. In contrast to the single nodular structures with a central AFB-containing necrotic core observed after infection with wild-type bacteria (Fig 5A1, 5A2, and 5A5), the lesions caused by mycolactone-deficient bacteria presented typically as multiple granulomatous structures densely grouped together (Fig 5B1). Several small central clusters of neutrophils were surrounded by an overall much larger proportion of infiltrating macrophages and T-cells (Fig 5B1–5B4 and 5B6) than in lesions caused by wild-type *M*. *ulcerans*. Fat cell ghosts were only observed in lesions caused by wild-type *M*. *ulcerans* (Fig 5A7) and the necrotic core was larger in those lesions compared to lesions caused by mycolactone-deficient bacteria, in which single granulomas did not contain a completely necrotic core (Fig 5A7 and 5B7).

### AFB in draining lymph nodes

**Fig 6:**
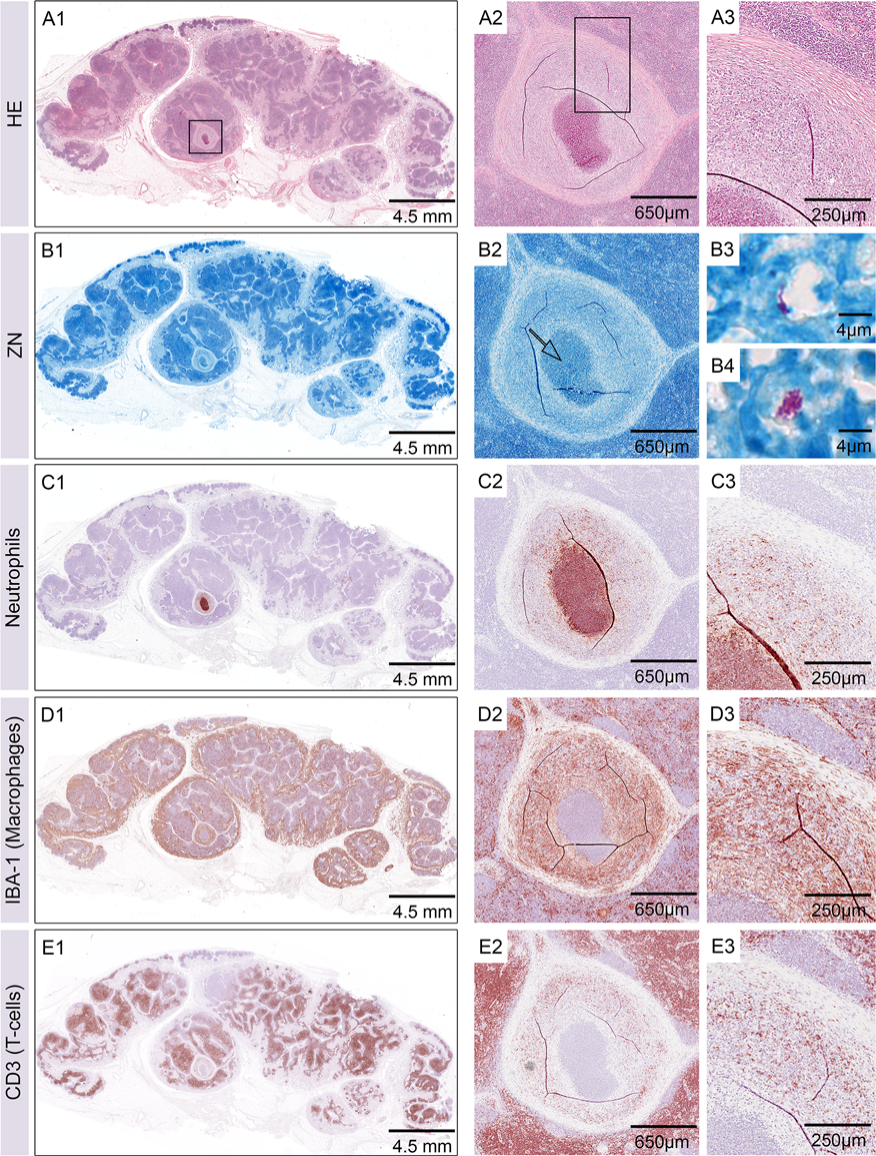
AFB in lymph nodes. Histological sections of one of the dorsal subiliac lymph nodes six weeks after infection. Haematoxylin-Eosin staining showed a small nodular structure inside the lymphoid tissue (A1-A3). ZN staining (B1-B4) confirmed the presence of AFB in the centre of the nodular structure (B2, arrow). Intact neutrophils (C1-C3) were directly associated with the AFB and surrounded by a belt of macrophages (D1-D3) that was interspersed with T-cells (E1-E3).

Additionally to the local infected skin we removed the three main lymph nodes (*Nll*. *cervicales superficiales ventrales* and *dorsales*, *Nll*. *subiliaci* [20]) draining the infected skin regions on each side of the pigs. Bacteria were found in one of the dorsal subiliac lymph nodes of the pig that had received injections with 1.3 x 10^6^ CFU *M*. *ulcerans* (Fig 6). A small structure of cellular infiltration was seen in the lymph node that consisted of a neutrophilic core without major necrosis. Similar to what we had observed in nodular lesions of the skin, this core was surrounded by a belt of macrophages that was interspersed with T-cells (Fig 6).

## Discussion

BU is most prevalent in rural regions of West African countries like Benin, Ghana, Cameroon and Ivory Coast, where patients often have only limited access to health care [23]. Together with the painless nature of the disease, patients often display late health care seeking behaviour [1]. In consequence, early non ulcerative stages of BU were rarely described histopathologically. Additionally, the number of tissue samples available for histopathological analyses has decreased tremendously, since the standard treatment consisting of surgical excision of the lesions was replaced with an eight-week antibiotic therapy with rifampicin and streptomycin. To contribute to the understanding of early host-pathogen interactions and to evaluate new treatment approaches and vaccines, we recently established the pig as a novel model for BU [16]. We demonstrated that infection of pigs with *M*. *ulcerans* induces lesions comparable to early BU lesions and we described the developing lesions by histopathology based on HE and ZN stainings.

In order to study the early local immune responses to *M*. *ulcerans* infection in more detail, we established and applied standard immunohistochemistry protocols for staining of porcine neutrophils, macrophages and T-cells in paraffin-embedded pig skin tissue sections. Although we failed to develop a method for staining of B-cells, cluster of B-cells, as also observed in treated human BU lesions [13], could still be identified. Despite the large number of antibodies specific for pig leukocyte antigens available, the number of antibodies suitable for staining paraffin-embedded tissue samples turned out to be very limited. Due to the fact that *in vivo* infection experiments with *M*. *ulcerans* have to be carried out under BSL-3 conditions in Switzerland, there was no alternative to formalin fixation and paraffin embedding of the tissue samples. With the three different cell types that we were able to stain, the main players in *M*. *ulcerans* infection, as known from the analysis of advanced human lesions [13], could be identified. However, a broader panel of antibodies against leukocyte markers for use in IHC on paraffin-embedded pig tissue would be of high interest.

To date only few human nodular lesions excised after antibiotic treatment have been analysed by immunohistochemistry [22]. They showed a similar cellular architecture to what we observed in the pig lesions six weeks after infection, with layers of infiltrating cells surrounding a necrotic core structure. Whether this layered structure can also be observed in human untreated BU nodules needs to be elucidated in future studies. However, the strong leukocyte infiltration at the site of infection in the pig lesions indicates that mycobacterial compounds are readily recognized by the innate immune system. The early immune response may in certain circumstances be capable of clearing the inoculum, a view that is supported by the results of serological studies that demonstrated that only a small proportion of individuals from BU endemic areas exposed to *M*. *ulcerans* develop BU disease [24,25]. Both the size of the inoculum and the yet not fully understood mode of transmission are likely to be of crucial importance for the outcome of an exposure to *M*. *ulcerans*. While subcutaneous inocula of 1.3 x 10^5^ and of 1.3 x 10^6^ CFU *M*. *ulcerans* were basically leading to the same pathogenic processes, we have shown previously, that after infection of pigs with 2 x 10^4^ CFU signs of infection found after 2.5 weeks had spontaneously resolved at 6.5 weeks [16]. With respect to the site of inoculation it has been shown in a guinea pig model that intradermal injection, but not topical application of *M*. *ulcerans* to abrasions leads to an establishment of an infection [26]. Human pre-ulcerative BU lesions may contain enormous amounts of AFB [8]. It is generally assumed that the major bacterial burden of these lesions is lost by ulceration, although this was never directly observed. This is now confirmed by our studies in pigs. While nodular lesions in the pig skin contained large amounts of AFB surrounded by layers of infiltrating leukocytes, the majority of *M*. *ulcerans* bacilli was no longer present in the ulcerated lesions. However, remains of AFB were detected in the crust on the ulcer and directly below in the upper dermis. Because we were mainly interested in studying early forms of BU lesions, we have so far not followed the progression of lesions to large ulcers. Therefore, we do not know whether the observed ulceration of the nodular lesions would lead to self-healing as it was recently reported in the guinea pig model [27] or whether similar to human lesions, AFB focally remaining in the undermined edges of the lesions would be sufficient to keep the disease on going.

Human and pig skin are strikingly similar [28] and our comparison of pig and human BU lesions supports a similar pathogenesis of the disease in the two species. Our observation in the pig skin that epidermal hyperplasia occurs only in close proximity to the lesion, is important for the analysis of human histopathological samples. Only small excisions or punch biopsies of the diseased tissue are usually available for diagnosis of human BU lesions by histopathology. Presence or absence of epidermal hyperplasia could be used as an estimate for the relative distance of the respective sample to the core of the lesion. The changes in the skin compared to healthy pig skin appear enormous considering an infection time of only six weeks, which might also be due to the high infection dose used. Further studies should focus on earlier infection time points and address the dynamics of the development of the lesions observed.

Infection with a mycolactone-negative *M*. *ulcerans* mutant led to a granulomatous reaction that was clearly different from the infiltration seen upon infection with wild-type *M*. *ulcerans*. The extent of cellular infiltration was larger and the AFB containing foci were not completely necrotic. Multiple small infection foci containing fewer and smaller clusters of AFB developed upon infection with the mycolactone-negative mutant. In contrast, infection with wild-type bacteria typically led to the development of one single infection focus containing multiple large AFB clusters. Despite these differences, the individual mutant-induced granulomas comprised the same sequential infiltration layers with central neutrophils surrounded by macrophages and T-cells. As expected, the mycobacterial antigen reservoir recognized by the innate immune system and leading to infiltration with different leukocytes thus seems to be essentially the same for wild-type and mutants. Mycolactone therefore appears to be the main factor responsible for the development of a central necrotic infection focus surrounded by layers of neutrophils, macrophages and lymphocytes.

Our data show that mycolactone-negative *M*. *ulcerans* are more effectively cleared from the site of infection than wild-type bacteria. The pathogenesis observed upon infection with mycolactone-negative *M*. *ulcerans* resembles that caused by *M*. *marinum* infection [29,30], which is primarily described both in fish and humans as a granulomatous disease. *M*. *ulcerans* developed from *M*. *marinum* by acquisition of a giant plasmid encoding for the enzymes necessary to produce myoclactone and has since drastically reduced its genome [31]. However, the evolutionary history appears to be still reflected in the response to infection, with mycolactone-negative *M*. *ulcerans* resembling more closely infection with *M*. *marinum* than infection with virulent *M*. *ulcerans*. Concerning the type of cellular infiltration occurring upon infection with toxin-negative mutants, results from experimental BU infections in the guinea pig [18] as well as the mouse model [32] are largely in line with what we observed in the pig model, except for the lack of neutrophil infiltration in guinea pig lesions. However, neutrophilic debris is present also in the necrotic centre of human BU lesions [8], which is indicative for an early wave of neutrophil infiltration in response to *M*. *ulcerans* infection.

Of the 16 dissected lymph nodes, only one contained AFB that were surrounded by neutrophils and macrophages. However, we cannot rule out, that more such micro-lesions were present in other lymph nodes, since we did not cut thin sections through the entire lymph nodes. It is unknown yet to what extent *M*. *ulcerans* is spreading to lymph nodes in BU patients, because lymph nodes adjacent to ulcerative lesions are usually not resected. The fact that the majority of BU patients present only a single lesion indicates that *M*. *ulcerans* is not readily spreading throughout the body, which is attributed to its temperature sensitivity [21].

In summary, the pathogenesis and early immune response to *M*. *ulcerans* infection in humans seems to be very well mirrored by the pig infection model. This makes the model an excellent tool for the final pre-clinical profiling of new treatment options and candidate vaccines.

## Supporting Information

S1 Fig Different layers of leukocyte infiltration in nodular lesions six weeks after infection. Histological section of a nodular lesion six weeks after infection with 1.3 x 10^6^ CFU of *M*. *ulcerans*. Ziehl-Neelsen/Methyleneblue (ZN) staining reveals a strong cellular infiltration around a central necrotic core containing AFB stained in pink (layer 1). The necrotic core (layer 1) is surrounded by three more layers (layers 2–4) of leucocytes differing in their cellular appearance. Optical definition of the layers was done as previously described [16].
(TIF)
