# Supplementary figures and images for: Local Cellular Immune Responses and Pathogenesis of Buruli Ulcer Lesions in the Experimental Mycobacterium Ulcerans Pig Infection Model
Source: PLoS Negl Trop Dis. 2016 Apr 29;10(4):e0004678. doi: 10.1371/journal.pntd.0004678 (PMC4851394; doi:10.1371/journal.pntd.0004678)

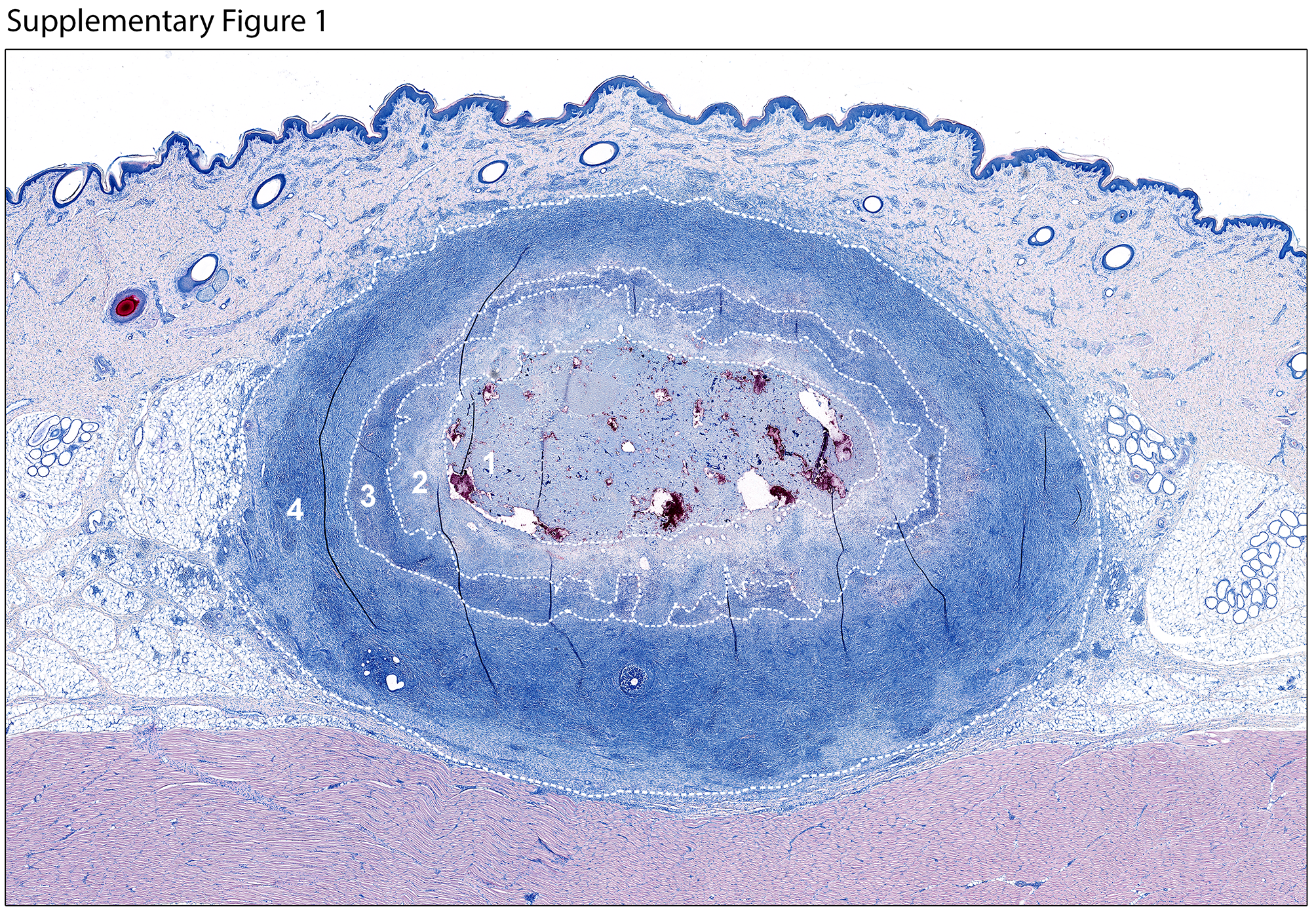

Supplement: S1 Fig — Histological section of a nodular lesion six weeks after infection with 1.3 x 10^6^ CFU of M. ulcerans. Ziehl-Neelsen/Methyleneblue (ZN) staining reveals a strong cellular infiltration around a central necrotic core containing AFB stained in pink (layer 1). The necrotic core (layer 1) is surrounded by three more layers (layers 2–4) of leucocytes differing in their cellular appearance. Optical definition of the layers was done as previously described [16]. (TIF) [file pntd.0004678.s001.tif]
